# A141 RISK OF TOTAL METACHRONOUS ADVANCED NEOPLASIA AFTER DETECTION OF PROXIMAL HYPERPLASTIC POLYPS, ADENOMAS, AND THEIR COMBINATION

**DOI:** 10.1093/jcag/gwad061.141

**Published:** 2024-02-14

**Authors:** W Safih, D von Renteln, I Popescu Crainic, C Haumesser, B Noyon, F Mubaid, C Rekkabi, P Marques, Y Li, R Djinbachian

**Affiliations:** Gastroenterology, Centre Hospitalier de l'Universite de Montreal, Montreal, QC, Canada; Gastroenterology, Centre Hospitalier de l'Universite de Montreal, Montreal, QC, Canada; Gastroenterology, Centre Hospitalier de l'Universite de Montreal, Montreal, QC, Canada; Gastroenterology, Centre Hospitalier de l'Universite de Montreal, Montreal, QC, Canada; Gastroenterology, Centre Hospitalier de l'Universite de Montreal, Montreal, QC, Canada; Gastroenterology, Centre Hospitalier de l'Universite de Montreal, Montreal, QC, Canada; Gastroenterology, Centre Hospitalier de l'Universite de Montreal, Montreal, QC, Canada; Gastroenterology, Centre Hospitalier de l'Universite de Montreal, Montreal, QC, Canada; Gastroenterology, Centre Hospitalier de l'Universite de Montreal, Montreal, QC, Canada; Gastroenterology, Centre Hospitalier de l'Universite de Montreal, Montreal, QC, Canada

## Abstract

**Background:**

Recent research findings have identified an association between proximal sessile serrated lesions (SSL) and a greater risk of advanced metachronous neoplasia, without significant impact of distal SSL.

**Aims:**

The principal aim of this study was to assess the risk of total metachronous advanced neoplasia (T-MAN) at the follow-up (fu) colonoscopy after detection of proximal hyperplastic polyps (HP), adenomas and their combination at the initial colonoscopy.

**Methods:**

Medical records for patients aged 45 to 74 who underwent colonoscopies in both 2014 and 2015 were reviewed. The primary outcome was the presence of T-MAN (comprising advanced adenomas or high-risk SL) during the follow-up colonoscopy depending on the presence of proximal HP, adenoma, or their combination at index colonoscopy. Secondary outcomes included assessing the risk of T-MAN at follow-up depending on index findings characteristics.

**Results:**

2014 patients were screened and 764 were included in the final analysis (44.1% male vs 55.9% female; mean age, 63y; median follow-up, 3.46 years). Patients with both proximal hyperplastic polyps and colonic adenomas during their initial colonoscopy had a significantly higher risk of developing T-MAN than patients with a combination of adenomas and distal HP or with only adenomas at index colonoscopy (30.5% vs 19%) [Hazard-ratio (HR)=1.95 (95% confidence interval (CI)1.3-2.9)]. A combination of proximal hyperplastic polyps and colonic adenomas is associated with a higher risk of T-MAN during follow-up than proximal HP alone (30.5% vs 13.9%) [HR=3.4 (95% CI 1.3-8.7)]. No statistically significant evidence was found to identify an increased risk of developing T-MAN at follow-up in patients presenting adenomas alone vs those with only proximal HP (19.1% vs 13.9%) [HR=1.9 (95% CI 0.75-4.7)].

**Conclusions:**

Patients with proximal hyperplastic polyps have an increased risk of presenting a T-MAN at the follow-up colonoscopy. Presenting a combination of hyperplastic polyps and adenomas indicates a higher risk of developing T-MAN than presenting an HP or adenoma alone.

**Funding Agencies:**

Daniel von Renteln is supported by a "Fonds de Recherche du Québec Santé" career development award. He has also received research funding from ERBE Elektromedizin GmbH, Ventage, Pendopharm, Fujifilm and Pentax, and has received consultant or speaker fees from Boston Scientific Inc., ERBE Elektromedizin GmbH, and Pendopharm. Roupen Djinbachian is supported by a “Fonds de Recherche du Québec Santé/Ministère de la Santé et des Services Sociaux” clinical research award. The remaining authors declare that they have no conflict of interest.

